# Co-Inhibition of BCL-W and BCL2 Restores Antiestrogen Sensitivity through BECN1 and Promotes an Autophagy-Associated Necrosis

**DOI:** 10.1371/journal.pone.0008604

**Published:** 2010-01-06

**Authors:** Anatasha C. Crawford, Rebecca B. Riggins, Ayesha N. Shajahan, Alan Zwart, Robert Clarke

**Affiliations:** Lombardi Comprehensive Cancer Center and Department of Oncology, School of Medicine, Georgetown University, Washington, District of Columbia, United States of America; Roswell Park Cancer Institute, United States of America

## Abstract

BCL2 family members affect cell fate decisions in breast cancer but the role of BCL-W (BCL2L2) is unknown. We now show the integrated roles of the antiapoptotic BCL-W and BCL2 in affecting responsiveness to the antiestrogen ICI 182,780 (ICI; Fulvestrant Faslodex), using both molecular (siRNA; shRNA) and pharmacologic (YC137) approaches in three breast cancer variants; MCF-7/LCC1 (ICI sensitive), MCF-7/LCC9 (ICI resistant), and LY2 (ICI resistant). YC137 inhibits BCL-W and BCL2 and restores ICI sensitivity in resistant cells. Co-inhibition of BCL-W and BCL2 is both necessary and sufficient to restore sensitivity to ICI, and explains mechanistically the action of YC137. These data implicate functional cooperation and/or redundancy in signaling between BCL-W and BCL2, and suggest that broad BCL2 family member inhibitors will have greater therapeutic value than targeting only individual proteins. Whereas ICI sensitive MCF-7/LCC1 cells undergo increased apoptosis in response to ICI following BCL-W±BCL2 co-inhibition, the consequent resensitization of resistant MCF-7/LCC9 and LY2 cells reflects increases in autophagy (LC3 cleavage; p62/SQSTM1 expression) and necrosis but not apoptosis or cell cycle arrest. Thus, *de novo* sensitive cells and resensitized resistant cells die through different mechanisms. Following BCL-W+BCL2 co-inhibition, suppression of functional autophagy by 3-methyladenine or BECN1 shRNA reduces ICI-induced necrosis but restores the ability of resistant cells to die through apoptosis. These data demonstrate the plasticity of cell fate mechanisms in breast cancer cells in the context of antiestrogen responsiveness. Restoration of ICI sensitivity in resistant cells appears to occur through an increase in autophagy-associated necrosis. BCL-W, BCL2, and BECN1 integrate important functions in determining antiestrogen responsiveness, and the presence of functional autophagy may influence the balance between apoptosis and necrosis.

## Introduction

Approximately 70% of all newly diagnosed breast cancers express estrogen receptor-alpha (ER) [1], many of which are sensitive to antiestrogens. The steroidal antiestrogen ICI 182,780 (ICI; Faslodex, Fulvestrant) is a selective ER downregulator (SERD) that acts as an ER antagonist and enhances ubiquitin-mediated ER degradation. ICI is an effective second-line treatment for TAM resistant, ER-positive (ER+) tumors, and is as effective as some aromatase inhibitors [2], [3]. One limitation of antiestrogen therapy is the prevalence of *de novo* and acquired resistance in breast cancer. Acquired antiestrogen resistance occurs when a tumor has an initially beneficial response to antiestrogen treatment but the remaining tumor cells stop responding [4], [5]. We report the roles of BCL2L2 (BCL-W), BCL2, and Beclin-1 (BECN1) in affecting responsiveness to ICI-resistance, and describe how anti-apoptotic BCL2 family members are involved in determining breast cancer cell fate.

BCL2 family proteins are essential regulators of apoptosis. BCL2 and BCL-W are both antiapoptotic members of this family. BCL-W maintains cell viability by preventing mitochondrial membrane depolarization and caspase activation [6]. BCL-W acts by binding to pro-apoptotic BCL2 family members and preventing mitochondria-mediated apoptosis [7]. Overexpression of BCL-W can prevent cell death [6] but its role(s) in affecting breast cancer cell fate decisions or antiestrogen responsiveness is unknown. BCL2 also blocks the induction of apoptosis by inhibiting the activation of pro-apoptotic family members such as BAX and preventing mitochondrial membrane depolarization [8], [9]. Overexpression of BCL2 is a potential mediator of resistance to several chemotherapeutic drugs [10].

BCL2 family members also play essential roles in autophagy (macroautophagy), a process characterized by the presence of autophagosomes that engulf damaged organelles for subsequent lysosomal degradation. Several anti-apoptotic BCL2 family members inhibit the activity of BECN1 [11], a key regulator of autophagy [12] that binds to PIK3C3 to facilitate autophagosome production [13]. However, the precise relationships between apoptosis and autophagy are unclear. Apoptosis or autophagy can each lead to cell death, but in some cellular contexts autophagy is a pro-survival process, for example, in the face of nutrient deprivation [11]. While autophagy can contribute to TAM resistance in some breast cancer cells [14]–[16], its role in response to other antiestrogens is unknown. In ER+ MCF-7 breast cancer cells treated with camptothecin, autophagy prolongs survival and delays apoptosis [17]. In marked contrast, autophagy promotes apoptosis in MCF-7 cells treated with the cytotoxic diterpenoid oridonin, where an inhibition of autophagy increases cell survival [18].

We determined whether BCL-W and BCL2 regulate ICI response in human breast cancer cells, and whether any effects involve changes in apoptosis and/or BECN1-associated autophagy. We used three estrogen-independent cell lines: MCF-7/LCC1 (ICI sensitive) [19], and LY2 and MCF-7/LCC9 cells that are crossresistant to TAM and ICI [20], [21]. We show that co-inhibition of BCL-W and BCL2 restores sensitivity to the growth-inhibitory effects of ICI in both MCF-7/LCC9 and LY2 cells. In re-sensitized cells, ICI treatment increases the levels of autophagy and necrosis but has no effect on apoptosis. Inhibition of autophagy by 3-methyladenine (3MA) or BECN1 shRNA under these conditions reduces necrosis and increases apoptosis. Thus, restoration of ICI sensitivity with BCL-W+BCL2 inhibition appears to occur through increasing an autophagy-associated necrotic cell death. Finally, we show that co-inhibiting BCL-W and BCL2 improves ICI sensitivity in antiestrogen-sensitive cells by increasing apoptosis. Therefore, BCL-W, BCL2, and BECN1 integrate central functions in determining ICI responsiveness likely by regulating functional autophagy to dictate the balance between apoptotic and necrotic cell death.

## Results

**Figure 1:**
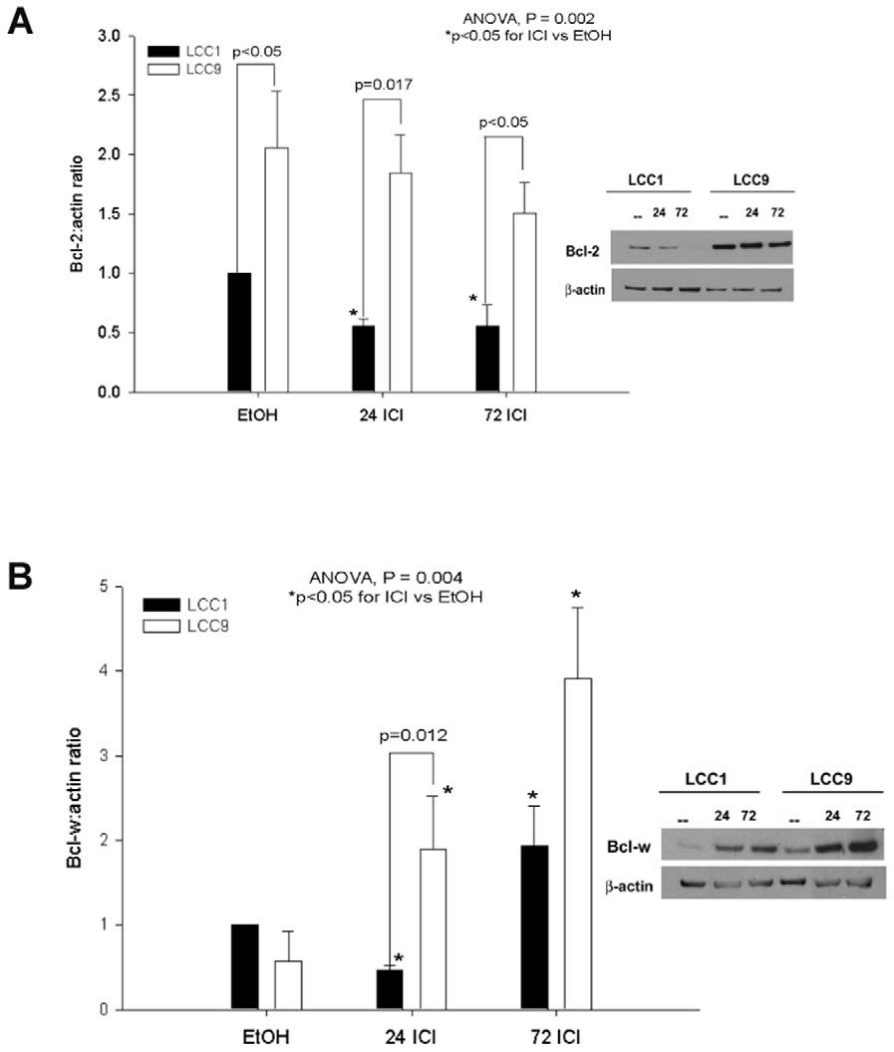
Increased expression of BCL-W and BCL2 in MCF-7/LCC9 cells. Whole cell lysates were subjected to Western blot analysis with a specific BCL2 or BCL-W antibody. (**A**) Bars represent the mean±SE of the relative BCL2∶actin ratio (normalized to control cells) for three independent experiments. *Inset*, *a representative blot*. (**B**) Bars represent the mean±SE of the relative BCL-W∶actin ratio (normalized to control cells) for three independent experiments. *Inset, a representative blot*.

We measured endogenous BCL-W and BCL2 expression in control and ICI treated resistant and sensitive cells. BCL2 expression was significantly higher in ethanol control and ICI treated (resistant) MCF-7/LCC9 cells when compared to (sensitive) MCF-7/LCC1 cells (**Figure 1A**; ANOVA p = 0.002). BCL-W expression was lower in MCF-7/LCC1 cells after 24 hr of ICI treatment and increased in both sensitive and resistant cells after 72 hr of ICI treatment. However, the levels in resistant cells remained higher than in sensitive cells (**Figure 1B**; ANOVA p = 0.004;).

To determine if BCL2 transcription is regulated in antiestrogen-resistant cells, we measured basal BCL2 promoter activity using a BCL2-luciferase promoter-reporter assay. Basal BCL2 promoter activity was increased 14-fold in MCF-7/LCC9 cells (**Figure S1**; p<0.003) when compared to MCF-7/LCC1 cells, suggesting that the transcriptional regulation of basal BCL2 expression is altered in MCF-7/LCC9 cells.

### YC137 Restores ICI 182,780 Sensitivity by Increasing Necrotic but Not Apoptotic Cell Death in Antiestrogen Resistant Cells

**Figure 2:**
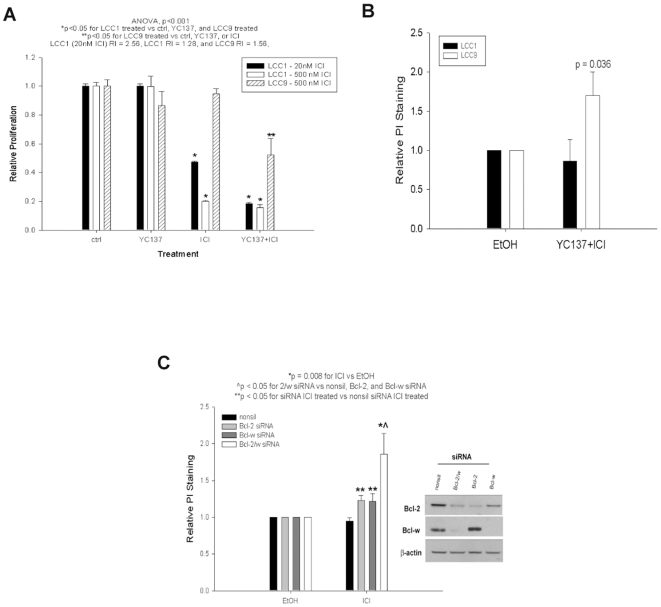
BCL-W and BCL2 inhibition increases sensitivity to ICI 182,780 and increases necrosis in MCF-7/LCC9 cells. (**A**) Cells were treated with YC137 and/or ICI for 7-days. Bars represent the mean±SE of relative cell proliferation (normalized to EtOH treated controls) for a single representative experiment performed in triplicate. (**B**) Cells were treated and stained with propidium iodide (PI). Bars represent the mean±SE of relative PI staining (normalized to control EtOH treated cells) for three independent experiments. (**C**) Cells were transfected with siRNA and stained with PI. *Inset, a representative blot showing BCL-W and BCL2 siRNA knockdown*.

We hypothesized that if the expression of pro-survival BCL2 family members is responsible for the resistance phenotype its inhibition should restore antiestrogen sensitivity. We first tested this hypothesis using the small molecule BCL2 inhibitor YC137 [22]. MCF-7/LCC1 and MCF-7/LCC9 cells were treated with YC137 (400 nmol/L) and ICI (20 nmol/L and 500 nmol/L) for 7-days. Total cell number was significantly decreased after treatment with both concentrations of ICI and/or YC137 in MCF-7/LCC1 cells (ANOVA p<0.001; Fig. 2*A*). RI = 2.56 (20 nmol/L ICI) and RI = 1.23 (500 nmol/L ICI) suggest a strong synergistic interaction between 20 nmol/L ICI and YC137; the weaker interaction between 500 nmol/L ICI and YC137 reflects the high potency of 500 nmol/L ICI alone in sensitive cells. In resistant cells, neither ICI nor YC137 alone affected cell proliferation, whereas total cell number decreased after YC137+ICI treatment (**Figure 2A**; ANOVA p = 0.001) indicating a restoration of ICI sensitivity; RI = 1.56 for the YC137+ICI treatment implies a synergistic interaction.

To determine the effect of BCL-W and BCL2 inhibition in sensitive cells, MCF-7/LCC1 cells were treated with increasing concentrations of YC137. Five days of YC137 treatment had no effect; however, cell proliferation decreased significantly after 7-days (**Figure S2A**; ANOVA p<0.001). After 5-days of YC137+ICI treatment, YC137 further decreased cell proliferation after treatment with ICI (**Figure S2B**; ANOVA p<0.001). Results with 20 nM ICI are included in Fig. 2*A* for comparison.

To determine if YC137 increases apoptosis, MCF-7/LCC1 and MCF-7/LCC9 cells were treated with YC137+ICI for 48 hr. Cell fate was evaluated by measuring FITC-Annexin V (apoptosis) and propidium iodide (PI) staining (to measure necrosis; not to detect the sub-G_1_ peak) by FACS. In contrast to MCF-7/LCC1 cells, treatment of MCF-7/LCC9 cells with YC137 or ICI only, or YC137+ICI did not induce apoptosis (not shown). However, in MCF-7/LCC9 cells treated with YC137+ICI a significant increase in PI staining was observed (**Figure 2B**; p = 0.036).

Whether the effects of YC137 are driven by inhibition of BCL-W, BCL2, or inhibition of both proteins is required, is unknown. To determine the effects of specific BCL2 family members on the changes in cell death seen with YC137 treated cells, BCL-W and BCL2 siRNA were used individually or concurrently to inhibit their expression. Knockdown of either BCL-W or BCL2 individually or in combination in MCF-7/LCC9 cells does not result in increased apoptosis when combined with ICI treatment in resistant cells (not shown). However, we detected a significant increase in PI staining after BCL-W±BCL2 knockdown and ICI treatment (**Figure 2C**; p<0.05), the greatest effect was seen when both BCL-W and BCL2 are co-inhibited (**Figure 2C**; p<0.05). To confirm these observations morphologically, cells were treated for 48 hr prior to staining with an acridine orange/ethidium bromide solution and examined by fluorescence microscopy. Images of viable cells (large, green nuclei), apoptotic cells (condensed, green nuclei), late apoptotic cells (condensed red nuclei), and necrotic cells (large, red-orange nuclei) were captured. The greatest proportion of necrotic cells is seen with YC137+ICI treatment (data not shown). These data show that BCL-W+BCL2 co-inhibition in ICI treated antiestrogen resistant cells most strongly increases necrosis without significantly altering the rate of apoptosis, while inhibition of BCL2 or BCL-W alone is not sufficient.

### ICI 182,780 Treatment Combined with BCL2 and BCL-W Inhibition Increases Autophagy in Resistant Cells

**Figure 3:**
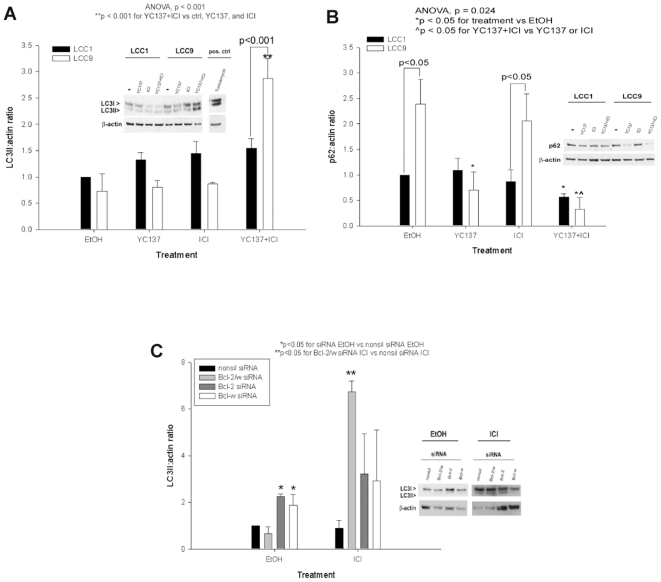
LC3II and p62/SQSTM1 expression after BCL-W and BCL2 inhibition. Whole cell lysates were subjected to Western blot analysis with a specific LC3 or p62/SQSTM1 antibody. (**A**) Bars represent the mean±SE of the relative LC3II∶actin ratio (normalized to empty vector controls) for three independent experiments. *Inset, a representative blot*. (**B**) Bars represent the mean±SE of the relative p62/SQSTM1∶actin ratio (normalized to empty vector controls) for three independent experiments. *Inset, a representative blot*. (**C**) Cells were transfected with siRNA and LC3II measured by Western blot analysis. Bars represent the mean±SE of the relative LC3II∶actin ratio (normalized to empty vector controls) for three independent experiments.

During autophagy LC3 is cleaved to form LC3I and LC3II, whereas p62/SQSTM1 binds to LC3 and is degraded [23]. To determine if YC137 treatment acts by increasing autophagy, as might be expected from its inhibition of BCL2 [24], cells were treated with YC137 and/or ICI and examined for LC3 cleavage and p62/SQSTM1 expression by Western blotting. In MCF-7/LCC9 cells, there was a significant increase in LC3II expression after YC137+ICI treatment when compared to ethanol treated controls and MCF-7/LCC1 cells (**Figure 3A**; ANOVA p<0.001). LC3II expression in the combination-treated cells was also significantly higher than in cells treated with either YC137 or ICI alone (**Figure 3A**; ANOVA p<0.001). Consistent with the predicted increase in autophagy, p62/SQSTM1 expression was downregulated in MCF-7/LCC9 cells treated with YC137 or YC137+ICI; expression in the combination treated cells was significantly lower than in cells treated with YC137 or ICI alone (**Figure 3B**; ANOVA p<0.024). This decrease in p62/SQSTM1 expression was also observed in YC137+ICI treated MCF-7/LCC1 cells (**Figure 3B**; ANOVA p<0.024). To determine if BCL2+BCL-W knockdown produces the same effect as YC137, MCF-7/LCC9 cells were transfected with both BCL2 and BCL-W siRNAs and treated with ICI. Consistent with the effects of YC137, BCL-W and BCL2 co-inhibition significantly increased LC3II expression after ICI treatment (**Figure 3C**; p<0.05).

### Combined Inhibition of BCL2, BCL-W, and Autophagy (by 3MA) Increases Apoptosis and Decreases Necrosis

**Figure 4:**
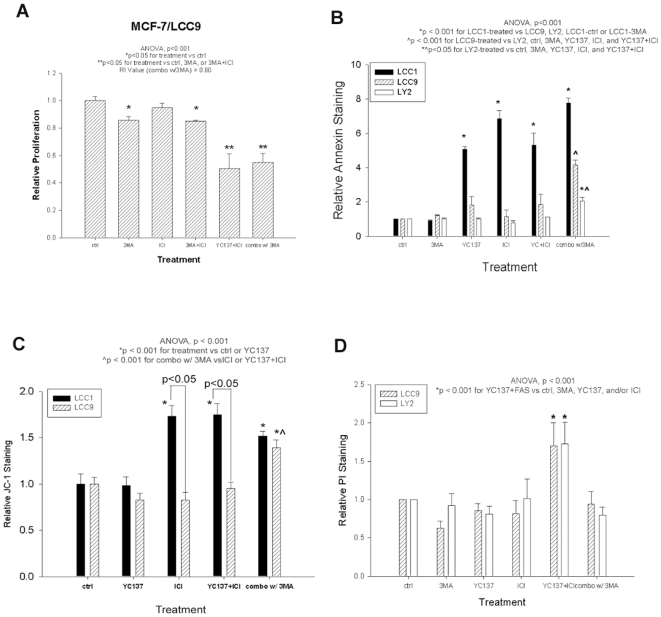
Increased apoptosis and decreased necrosis after BCL-W and BCL2 and autophagy inhibition. (**A**) Cells were treated with ICI, 3MA, YC137, or a combination of the three for 7-days. Bars represent the mean±SE of relative proliferation (normalized to empty vector control). (**B**) Cells were treated with YC137, ICI, 3MA, YC137+ICI, or a combination of YC137, ICI, and 3MA for prior to Annexin V staining. Bars represent the mean±SE of the relative Annexin V staining (normalized to empty vector controls) for three independent experiments. (**C**) Cells were treated with ICI, 3MA, YC137, or a combination prior to JC-1 staining. (**D**) Cells were treated with YC137, ICI, 3MA, YC137+ICI, or a combination of YC137, ICI, and 3MA prior to PI staining. Bars represent the mean±SE of the relative PI staining (normalized to empty vector controls) for three independent experiments.

To investigate the functional role of autophagy after BCL-W and BCL2 co-inhibition, MCF-7/LCC9 cells were treated with the autophagy inhibitor 3MA (350 µmol/L) in combination with ICI and YC137. Cell number was significantly decreased in 3MA+ICI+YC137 co-treated cells (**Figure 4A**; ANOVA p<0.001). However, treatment with 3MA+ICI+YC137 did not decrease further MCF-7/LCC9 cell proliferation when compared to ICI+YC137 (**Figure 4A**).

Autophagy can be pro-death [15], [18] or pro-survival [17], [25]. To determine the effect of autophagy inhibition on cell death, we measured mitochondrial membrane permeability (MMP), apoptosis, and necrosis after treatment with 3MA. The ICI-resistant LY2 cells were also examined to compare their response to BCL2+BCL-W co-inhibition and autophagy inhibition with ICI-resistant MCF-7/LCC9 cells. While LY2 cells express low basal levels of BCL-W, BCL2, and LC3II (not shown), cell proliferation was significantly down-regulated following YC137, ICI, and YC137+ICI treatment; proliferation is lowest in combination treated cells when compared to the individual treatments (**Figure S3**; ANOVA, p<0.001). Following treatment with 3MA+YC137+ICI, Annexin V staining increased significantly in resistant cells (MCF-7/LCC9; LY2) when compared to controls, cells treated with each of 3MA, YC137, or ICI alone, or YC137+ICI (**Figure 4B**; ANOVA p<0.001). MCF-7/LCC1 cells increased relative Annexin V staining after all treatments except when treated with 3MA alone (**Figure 4B**; p<0.001).

We then determined if these effects were associated with changes in the mitochondria. Consistent with the Annexin V staining, MMP increased significantly in MCF-7/LCC1 cells following treatment with ICI and YC137 alone, and after treatment with ICI combined with YC137 and/or 3MA. In contrast, MCF-7/LCC9 cells exhibit increased MMP only after treatment with 3MA+YC137+ICI (**Figure 4C**; p<0.001). A decrease in PI staining, indicating a decrease in necrosis, occurred in resistant cells only after the addition of 3MA to YC137+ICI (**Figure 4D**; ANOVA p<0.001). There was no change in the number of cells in S-phase after treatment of MCF-7/LCC9 cells with YC137, 3MA, YC137+ICI, or 3MA+YC137+ICI (**Figure S4**). Thus, the reversal of resistance can occur without the cell cycle arrest seen in *de novo* sensitive cells. As expected, the number of MCF-7/LCC1 cells undergoing S-phase decreased after YC137+ICI and 3MA+YC137+ICI treatment (ANOVA, p = 0.016; **Figure S4**).

**Figure 5:**
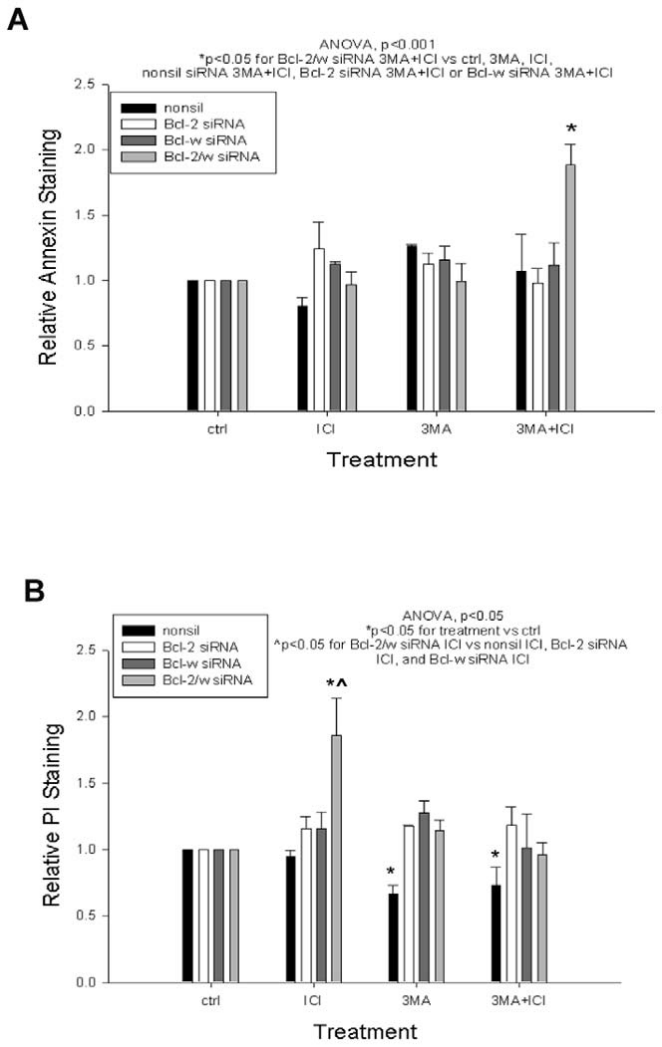
BCL-W/BCL2 knockdown and autophagy inhibition increases apoptosis and decreases necrosis. (**A**) MCF-7/LCC9 cells were transfected with a combination of BCL-W and BCL2 siRNA and treated with ICI and 3MA. Bars represent the mean±SE of the relative Annexin V staining (normalized to empty vector controls) for three independent experiments. (**B**) Bars represent the mean±SE of the relative PI staining (normalized to empty vector controls) for three independent experiments.

To establish further the roles of BCL-W and BCL2, we performed similar studies in BCL-W+BCL2 siRNA co-transfected resistant cells (MCF7/LCC9; LY2) treated with ICI and/or 3MA. After treating the siRNA transfected MCF-7/LCC9 cells with a combination of ICI and 3MA, we found a significant increase in Annexin V staining in BCL-W and BCL2 siRNA co-transfected cells (**Figure 5A**; ANOVA p<0.001). A significant decrease in PI staining was also observed after BCL-W/BCL2 knockdown in combination with ICI+3MA treatment (**Figure 5B**; ANOVA p = 0.05). These data imply that functional autophagy plays a major role in influencing the decision to undergo apoptosis and/or necrosis in antiestrogen-resistant cells.

### BECN1 Mediates Key Effects of YC137, BCL-W, and BCL2

**Figure 6:**
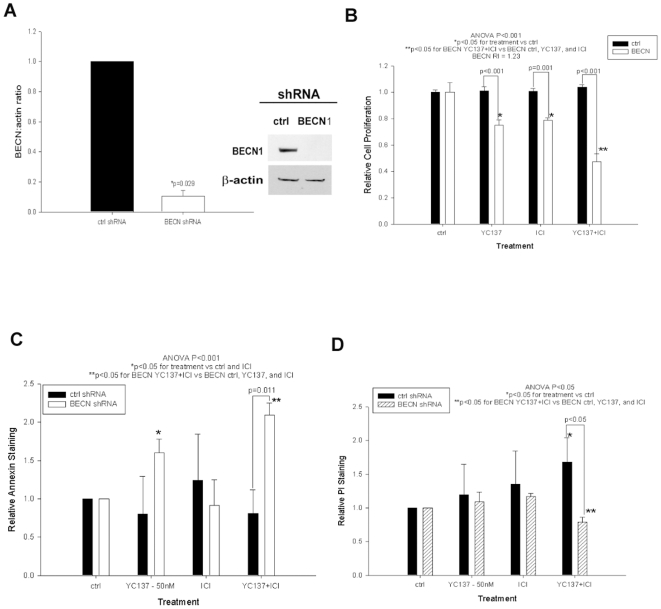
BECN1 knockdown and BCL-W/BCL2 co-inhibition decreases cell proliferation through increased apoptosis in resistant cells. (**A**) Whole cell lysates were subjected to Western blot analysis with a specific BECN1 monoclonal antibody. Bars represent the mean±SE of the relative BECN1∶actin ratio (normalized to control cells) for three independent experiments. (**B**) shRNA infected MCF-7/LCC9 cells were treated with YC137 and/or ICI for 7-days. Bars represent the mean±SE of relative cell proliferation (normalized to EtOH treated controls) for a single representative experiment performed in triplicate. (**C**) shRNA infected MCF-7/LCC9 cells were treated with YC137, ICI, or a combination of YC137 and ICI. Bars represent the mean±SE of the relative Annexin V staining (normalized to empty vector controls) for three independent experiments. (**D**) Bars represent the mean±SE of the relative PI staining (normalized to empty vector controls) for three independent experiments.

While basal BECN1 expression levels are comparable in sensitive and resistant cells (not shown), we could not exclude the possibility that its role is functionally different in these cellular contexts. Thus, we explored the mechanistic relationship between BCL-W, BCL2, and BECN1 using shRNA-mediated knockdown of BECN1 in resistant cells. BECN1 shRNA effectively decreased BECN1 protein expression (approximate 5-fold) in MCF-7/LCC9 cells (**Figure 6A**; p = 0.029). Also, BECN1 shRNA infected MCF-7/LCC9 cells were more sensitive to YC137 (50 nmol/L) and ICI (500 nmol/L) than control infected cells, and cell proliferation was down-regulated after treatment with YC137 or ICI (**Figure 6B**; ANOVA, p<0.001). However, cell proliferation following YC137+ICI treatment was significantly lower than either treatment alone. For BECN1 knockdown combined with ICI treatment, RI = 1.23 suggests at least an additive interaction. Unlike control infected cells, proliferation was downregulated in BECN1 shRNA infected cells treated with YC137 or YC137+ICI (**Figure 6B**; ANOVA p<0.001). Furthermore, apoptosis was significantly increased in cells treated with YC137+ICI (**Figure 6C**; ANOVA p<0.001). The level of necrosis increased in control infected cells, and decreased in BECN1 shRNA infected cells, when treated with YC137+ICI (**Figure 6D**; ANOVA p<0.05). BECN1 knockdown, in combination with BCL2+BCL-W co-inhibition, inhibited autophagy, restored ICI sensitivity, and increased apoptosis (but not necrosis) in ICI treated antiestrogen-resistant breast cancer cells. These different cell death outcomes in sensitive and resistant cells indicate considerable plasticity in breast cancer cell fate mechanisms in response to antiestrogens.

## Discussion

Antiestrogen resistance is a major limitation to improving breast cancer survival rates and elucidating its mechanisms remains an important challenge [26], [27]. In breast tumors, BCL2 expression measured prior to therapy correlates with ER expression and an improved response to antiestrogens [28]. However, BCL2 levels decrease after TAM therapy, but only in those women who obtain clinical benefit [29]. In breast tumors, apoptosis increases after the first 24 hr of TAM treatment but markedly decreases 3-months later. Moreover, BCL2 expression is elevated in residual (resistant) tumors [30]. We hypothesized that increased expression of BCL2 and/or BCL-W may play a role in antiestrogen resistance by allowing resistant cells to evade apoptosis. We show that in the absence of estrogen there is an increase in basal and ICI-regulated BCL2 mRNA, protein, and promoter activity in resistant cells, observations consistent with data showing elevated activity of two upstream regulators of BCL2: NFκB and XBP1 [31]–[33]. However, co-inhibition of BCL-W and BCL2 is required to restore ICI sensitivity, a process that is driven by increased autophagy and necrosis, but not apoptosis. We also show that increased autophagy may activate necrotic cell death in resistant cells.

Little is known about BCL-W expression and function in breast cancer. Since BCL-W is overexpressed in some human colon cancer cells [6], [34] and its expression is regulated by estrogen in cerebrocortical neuron cultures [35], we hypothesized that BCL-W could play a role in antiestrogen resistance. BCL-W expression is increased by ICI in both sensitive and resistant cells, suggesting that an increased co-expression of both BCL2 and BCL-W is required for antiestrogen resistance. Hence, the ICI-induced increase in the expression of BCL-W alone in antiestrogen-sensitive cells has little effect on responsiveness unless accompanied by a concurrent increase in BCL2, as is seen in resistant cells.

Small-molecule inhibitors of proapoptotic BCL2 family members can restore sensitivity to some therapeutic agents that induce apoptosis [36]. Some of these compounds inhibit the proliferation of cells that express high levels of BCL2 [37]. However, several antiapoptotic BCL2 family members also regulate autophagy through their interactions with BECN1 [12], [38], [39]. In resistant MCF-7/LCC9 and LY2 cells, only the levels of autophagy and necrosis increase after YC137+ICI treatment; there is no increase in either MMP or apoptosis.

We used BCL-W and/or BCL2 siRNA to confirm the results with YC137. As expected, BCL-W+BCL2 co-inhibition has no effect on apoptosis in ICI treated MCF-7/LCC9 cells, whereas both autophagy and necrosis increase. Inhibition of BCL2 and BCL-xL decreases cellular ATP and increases necrosis (but not apoptosis) in acinar cells hyperstimulated with CCK-8 [40]. Autophagy can also activate necrosis in apoptosis-deficient mouse embryonic fibroblasts [41]. Increased autophagosome formation is induced early during necrotic cell death and contributes to the cellular destruction that occurs during necrosis in *Caenorhabditis elegans* [42]. These results suggest that BCL-W+BCL2 coinhibition can increase antiestrogen sensitivity in resistant breast cancer cells by preferentially activating necrosis, apparently in association with the induction of autophagy. In contrast, inhibiting autophagy in some TAM-resistant breast cancer cells can increase apoptosis [16].

No change occurs in the proportion of cells undergoing S-phase after 3MA+YC137+ICI treatment. Thus, it is unlikely that autophagy plays a major role in the cell cycle arrest effects of antiestrogens. We also show that the inhibition of autophagy, in combination with BCL-W+BCL2 co-inhibition in ICI treated resistant cells, does not further reduce total cell number but shifts programmed cell death such that apoptosis increases and necrosis decreases. Our results strongly suggest that functional autophagy is a central component of the cell fate decision machinery in ICI-resistant breast cancer cells, although we cannot exclude the possibility that autophagy also alters the kinetics of cell death. Nonetheless, in addition to being a cell death effector mechanism, autophagy appears to be a central component in influencing how breast cancer cells die in response to antiestrogens.

In summary, our results show that BCL-W+BCL2 co-inhibition restores ICI sensitivity in antiestrogen-resistant cells and increases ICI sensitivity in antiestrogen-sensitive cells. We show that the overexpression of BCL-W and BCL2 is linked to determining cell fate through autophagy in ICI resistant breast cancer models (**Figure S5A**). We have shown that BCL-W+BCL2 coinhibition increases autophagy and necrosis with no effect on the extent of apoptotic cell death (**Figure S5B**). These data suggest that BCL-W and BCL2 activate apoptosis and necrosis by initially regulating autophagy (**Figure S5C**). We conclude that the co-inhibition of BCL-W and BCL2 restores sensitivity in antiestrogen-resistant breast cancer cells by promoting an autophagy-associated increase in necrosis. Antiestrogen sensitive cells undergo autophagy and/or apoptosis, whereas resistant cells undergo autophagy and necrosis when resensitized. These different cell death outcomes in sensitive and resistant cells show the notable plasticity of cell fate mechanisms in breast cancer. In resistant cells, resensitization to antiestrogens can also occur without the cell cycle arrest that accompanies cell death in *de novo* sensitive cells. Thus, antiestrogen-regulated signaling that modifies cell cycling occurs through mechanisms independent of mitochondrial function and cell death.

From a therapeutic perspective, these data also suggest that broad rather than specific BCL2 family member inhibitors will have greater clinical value and may explain the apparent lack of activity of targeted BCL2 antisense monotherapy in clinical trials [43]. Combination therapy with endocrine agents and broadly active small molecule inhibitors of BCL2 family members may delay, prevent, or reverse the acquisition of antiestrogen resistance in breast cancer patients and lead to significant improvements in survival.

## Materials and Methods

### Cell Culture

All cells were shown to be free of *Mycoplasma* spp. contamination. MCF-7/LCC1 (ER+, estrogen independent, antiestrogen-sensitive) [19]; MCF-7/LCC9 (ER+, estrogen independent, TAM and ICI cross-resistant variant derived from MCF-7/LCC1 cells by selection against ICI) [21], and LY2 cells (ER+, estrogen independent, LY 117018, TAM, and ICI cross-resistant, MCF-7 variant derived by selection against the Raloxifene analog LY 117018) [20] were routinely grown in improved minimal essential medium without phenol red and supplemented with 5% charcoal stripped calf serum (CCS-IMEM; Biofluids). We confirmed the genetic lineage of the three variant cell lines as being derived from the original MCF-7 cell line by DNA fingerprinting using genetic markers at nine different loci. All cells were maintained at 37°C in a humidified incubator with 95% air∶5% CO_2_ atmosphere. ICI was obtained from Tocris Bioscience (Ellisville, MO) and 3-methyladenine (3MA) from Sigma Aldrich (St. Louis, MO). Acridine orange was obtained from EMD Biosciences (San Diego, CA) and ethidium bromide from Invitrogen (Carlsbad, CA). YC137 was kindly provided by Dr. York Tomita (Georgetown University) [44].

### RNA Isolation and Quantitative Real-Time PCR

Total RNA was isolated using the Trizol method. For each cDNA sample a qPCR reaction and a standard curve were established using TaqMan Universal PCR Master Mix and the following TaqMan primers (Applied Biosystems): BCL2 = Hs00608023_m1; BCL-W (BCL2L2) = Hs00187848_m1; RPLP0 (housekeeping gene) = Hs99999902_m1. Each reaction (10 µl) was run in triplicate on an ABI Prism 7900HT Sequence Detection System using the manufacturer's absolute quantification protocol. Expression data for each reaction was estimated relative to expression of RPLP0.

### Transient Transfection and Promoter-Reporter Assays

Cells were plated at 60,000 cells/well and maintained for 24 hr prior to co-transfection with 0.4 µg of full length BCL2 promoter-luciferase reporter plasmid [45] (a generous gift from Dr. Linda Boxer, Stanford University Medical Center) and 0.004 µg of the phRL-SV40-Renilla control plasmid containing the *Renilla* luciferase gene (Promega, Madison, WI). Activation of the BCL2 promoter was measured using the Dual Luciferase Assay Kit (Promega) and luminescence measured using a Lumat LB 9501 luminator (EG&G Berthold, Bundoora, Australia).

### siRNA Transfection and Lentiviral shRNA Infection

Cells were plated at 100,000 cells/well and BCL2, BCL-W (Dharmacon, Lafayette, CO), and control siRNA (Santa Cruz Biotechnology, Santa Cruz, CA) were each diluted to 100 nM. Transfection was performed according to Dharmacon's protocol using Lipofectamine 2000 (Invitrogen). Twenty-four hours after transfection, cells were treated with ICI, 3MA, a combination of the two, or ethanol vehicle for 48 hr. For the lentiviral infection, cells were plated at 10,000 cells/well and allowed to incubate for 24 hr prior to shRNA infection. BECN1 lentiviral particles and control lentiviral particles were purchased from Dharmacon. The infection was carried out according to the Dharmacon SMARTvector shRNA lentiviral protocol using Polybrene (Millipore).

### Western Blotting

Cells were treated as appropriate and lysed in radioimmunoprecipitation assay buffer [150 mmol/L NaCl, 50 mmol/L Tris (pH 7.5), 1% Igepal CA-630, and 0.5% deoxycholate] supplemented with Complete Mini protease inhibitor cocktail tablets (Roche) and 1 mmol/L sodium orthovanadate phosphatase inhibitor (Sigma). The primary antibodies used were: mouse monoclonal BCL2 primary antibody (1∶1000; Assay Designs, Ann Arbor, MI), rabbit monoclonal BCL-W primary antibody (1∶500; Cell Signaling, Danvers, MA), rabbit polyclonal LC3B primary antibody (1∶500; Cell Signaling), mouse monoclonal p62/SQSTM1 primary antibody (1∶500; Abcam, Cambridge, MA) overnight. Antigen-antibody complexes were visualized using the ECL detection system (Amersham Biosciences) and SuperSignal Chemiluminescent Substrate (Thermoscientific). Protein expression was quantified using densitometric analysis; data (mean±SE) are presented as the ratio of target protein∶βactin signals.

### Cell Proliferation

5,000 cells/well were treated as appropriate for 7-days. Following treatment, cells were stained with a crystal violet staining solution [46]. Sodium citrate buffer was added to each well and absorbance measured at 550 nM using a microplate reader (Biorad, Hercules, CA).

### Cell Cycle, Apoptosis, Necrosis, and Autophagy

Fluorescence activated cell sorting (FACS) was performed by the Lombardi Comprehensive Cancer Center Flow Cytometry Shared Resource. For cell cycle analysis, cells were plated at 80,000–100,000 cells/well, treated as appropriate for 48 hr, fixed, and analyzed by FACS. To measure apoptosis, cells were treated for 48 hr and stained as described in the TACS Annexin V Kit (Trevigen, Gaithersburg, MD). Necrosis was measured by counting cells stained red by propidium iodide (PI). For morphologic analysis of necrosis, cells were plated, treated 24 hr later, and after a further 48 hr stained with acridine orange/ethidium bromide solution (100 µg/ml acridine orange in PBS∶100 µg/ml ethidium bromide in PBS) and examined using an Olympus IX-70 confocal microscope with 488 nm and 633 nm excitation lasers.

To measure autophagy, we performed Western blot analysis to measure LC3 cleavage and p62/SQSTM1 expression [23]. Cells treated with 2 µg/ml tunicamycin (EMD Biosciences) for 48 hours were the positive control for LC3 cleavage. To block functional autophagy, we treated cells with the autophagy inhibitor 3MA, or infected cells with lentiviral BECN1 shRNA.

### Mitochondrial Membrane Permeability

Cells were treated as appropriate and stained with 100 µl of JC-1 dye solution (Invitrogen) for 25 min at 37°C. Green fluorescence (485 nm excitation/535 nm emission) was measured on a Wallac Viktor2 1420 Multilabel Counter (Perkin-Elmer, Boston, MA).

### Statistical Analyses

One-way ANOVA was used to determine overall significant differences following treatment in the cell proliferation, cell cycle, apoptosis, and MMP assays. Student's t-test was used to determine differences in BCL2, BCL-W, LC3, p62/SQSTM1 expression and luciferase promoter-reporter activity. All statistical analyses were performed using SigmaStat version 3.0. The nature of drug interactions (synergy, antagonism, additivity) was assessed using the Relative Index (RI) [47]. RI values were obtained by calculating the expected cell survival (S_exp_; the product of survival obtained with drug A alone and the survival obtained with drug B alone) and dividing this S_exp_ by the observed cell survival in the presence of both drugs (S_obs_). S_exp_/S_obs_>1.0 indicates a synergistic interaction, <1.0 indicates an antagonistic interaction, and  = 1 is indicative of an additive interaction between the two drugs used.

## Supporting Information

Figure S1 Increased basal BCL2 promoter activity in ICI/TAM-cross-resistant MCF-7/LCC9 cells. Cells were seeded in 12-well plates and co-transfected with BCL2 promoter-luciferase and pCMV-Renilla constructs for 24 h prior to lysis and luminescent detection (to examine basal promoter activity). Bars represent the mean±SE of the relative BCL2-luciferase: Renilla luciferase activity for a single representative experiment performed in triplicate. p<0.003 for MCF-7/LCC9 vs. MCF-7/LCC1.
(1.44 MB TIF)

Figure S2 Increased sensitivity to ICI 182,780 in antiestrogen-sensitive cells. A, MCF-7/LCC1 cells were treated with the indicated concentrations of YC137 for 5 and 7 days, at which time cell number was determined. Points represent the mean±SE of relative proliferation (normalized to empty vector control). ANOVA p<0.001; p<0.05 for YC137 vs. control. B, MCF-7/LCC1 cells were treated with ICI or a combination of YC137+ICI for 5 days, at which time cell number was determined. Points represent the mean±SE of relative proliferation (normalized to empty vector control). ANOVA p<0.001; p<0.001 for YC137+ICI treated cells vs. ICI treated cells.
(1.01 MB TIF)

Figure S3 BCL-W/BCL2 inhibition decreases cell proliferation in ICI 182,780 treated resistant LY2 cells. Cells were treated with ICI, YC137, or a combination of the two for 7 days to examine cell proliferation. Bars represent the mean±SE of relative proliferation (normalized to empty vector control). ANOVA p<0.001; p<0.05 for treatment vs. control treated cells and for YC137+ICI treated cells vs. YC137 and ICI.
(0.66 MB TIF)

Figure S4 Combined autophagy inhibition and BCL-W/BCL-2 inhibition does not alter cell cycle distribution in the resistant cell line. Cells were treated with ICI, 3MA, YC137, or a combination for 48 h prior to ethanol fixation and FACS analysis. ANOVA p = 0.016; p<0.05 for treatment vs. control, 3MA, or YC137.
(0.56 MB TIF)

Figure S5 BCL-W and BCL2 indirectly regulate necrosis through the direct regulation of autophagy and apoptosis. A, Representation of the relationship between BCL-W/BCL2 overexpression, autophagy, necrosis, and apoptosis in ICI-resistant cells treated with ICI 182,780. B, Representation of the effect of BCL-W/BCL2 inhibition on autophagy, necrosis, and apoptosis. C, Representation of the effect of BCL-W/BCL2 inhibition in combination with autophagy inhibition on autophagy, necrosis, and apoptosis.
(0.16 MB TIF)
