# Supplementary figures and images for: Co-Inhibition of BCL-W and BCL2 Restores Antiestrogen Sensitivity through BECN1 and Promotes an Autophagy-Associated Necrosis
Source: PLoS One. 2010 Jan 6;5(1):e8604. doi: 10.1371/journal.pone.0008604 (PMC2797635; doi:10.1371/journal.pone.0008604)

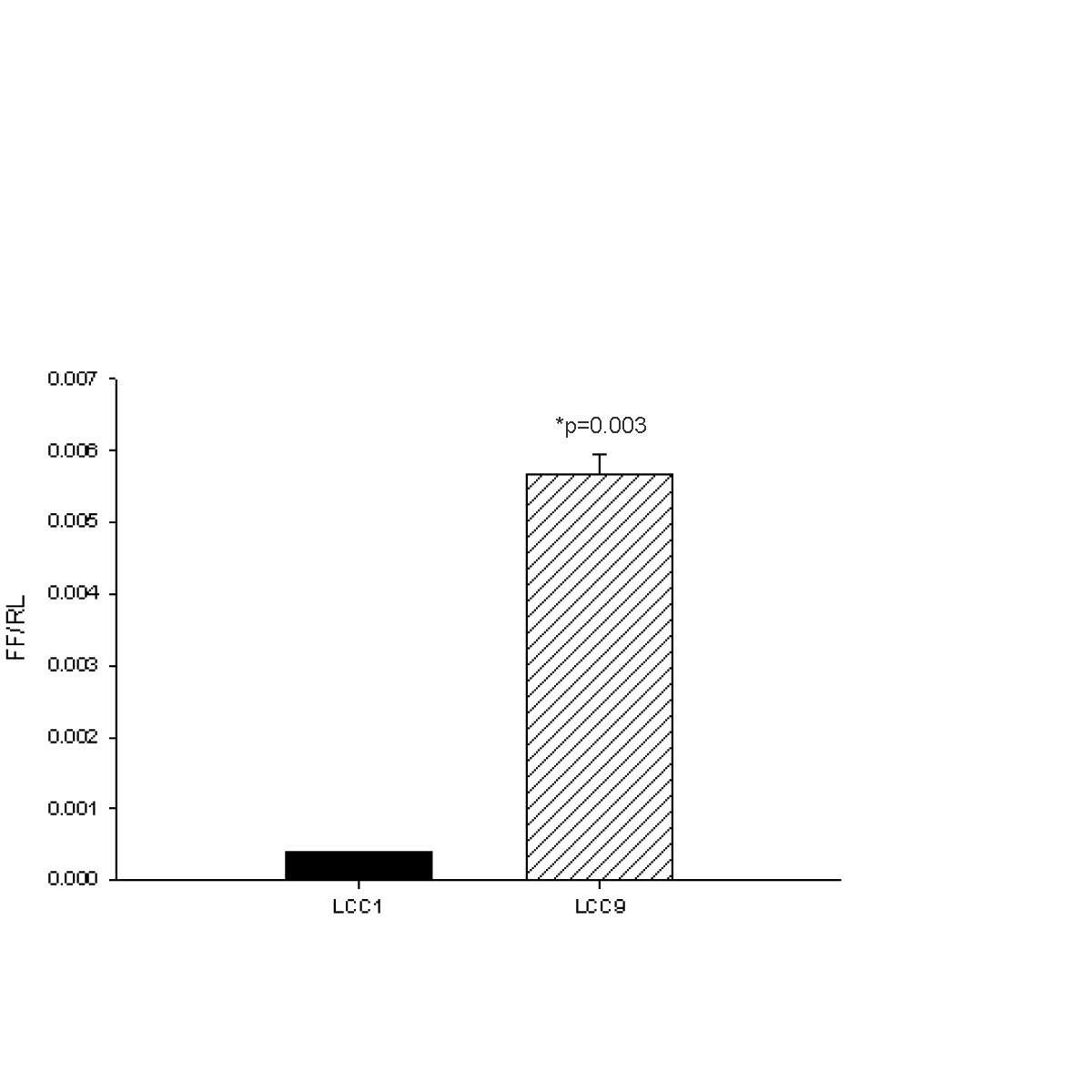

Supplement: Figure S1 — Increased basal BCL2 promoter activity in ICI/TAM-cross-resistant MCF-7/LCC9 cells. Cells were seeded in 12-well plates and co-transfected with BCL2 promoter-luciferase and pCMV-Renilla constructs for 24 h prior to lysis and luminescent detection (to examine basal promoter activity). Bars represent the mean±SE of the relative BCL2-luciferase: Renilla luciferase activity for a single representative experiment performed in triplicate. p<0.003 for MCF-7/LCC9 vs. MCF-7/LCC1. (1.44 MB TIF) [file pone.0008604.s001.tif]

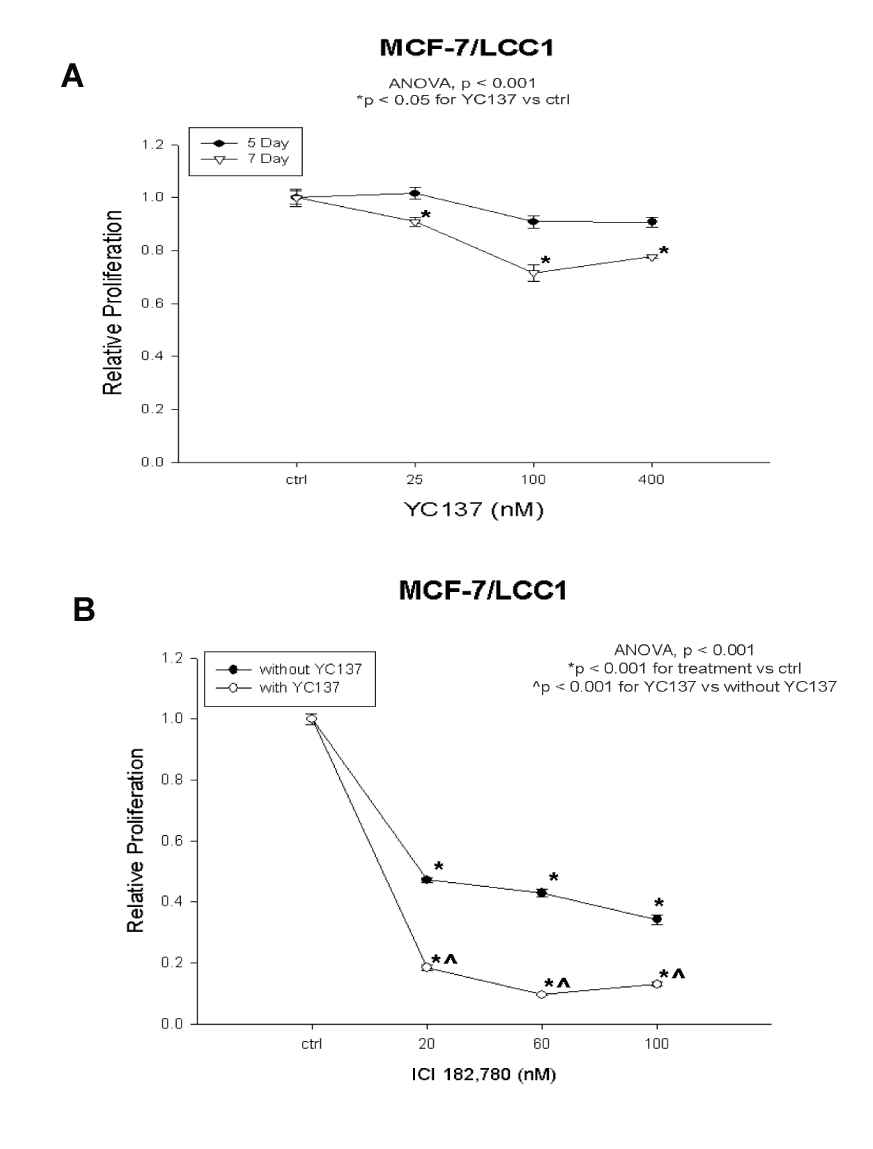

Supplement: Figure S2 — Increased sensitivity to ICI 182,780 in antiestrogen-sensitive cells. A, MCF-7/LCC1 cells were treated with the indicated concentrations of YC137 for 5 and 7 days, at which time cell number was determined. Points represent the mean±SE of relative proliferation (normalized to empty vector control). ANOVA p<0.001; p<0.05 for YC137 vs. control. B, MCF-7/LCC1 cells were treated with ICI or a combination of YC137+ICI for 5 days, at which time cell number was determined. Points represent the mean±SE of relative proliferation (normalized to empty vector control). ANOVA p<0.001; p<0.001 for YC137+ICI treated cells vs. ICI treated cells. (1.01 MB TIF) [file pone.0008604.s002.tif]

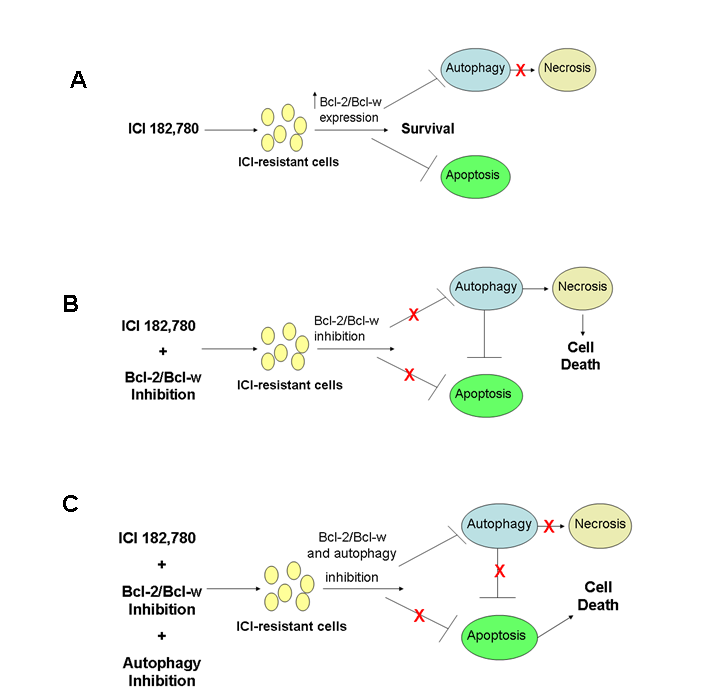

Supplement: Figure S5 — BCL-W and BCL2 indirectly regulate necrosis through the direct regulation of autophagy and apoptosis. A, Representation of the relationship between BCL-W/BCL2 overexpression, autophagy, necrosis, and apoptosis in ICI-resistant cells treated with ICI 182,780. B, Representation of the effect of BCL-W/BCL2 inhibition on autophagy, necrosis, and apoptosis. C, Representation of the effect of BCL-W/BCL2 inhibition in combination with autophagy inhibition on autophagy, necrosis, and apoptosis. (0.16 MB TIF) [file pone.0008604.s005.tif]
